# New Carbapenemase Inhibitors: Clearing the Way for the β-Lactams

**DOI:** 10.3390/ijms21239308

**Published:** 2020-12-06

**Authors:** Juan C. Vázquez-Ucha, Jorge Arca-Suárez, Germán Bou, Alejandro Beceiro

**Affiliations:** Servicio de Microbiología, Instituto de Investigación Biomédica de A Coruña (INIBIC-CICA), Complejo Hospitalario Universitario A Coruña (CHUAC), As Xubias 84, 15006 A Coruña, Spain; juan.vazquez@udc.es (J.C.V.-U.); jorge.ar.su@gmail.com (J.A.-S.); German.Bou.Arevalo@sergas.es (G.B.)

**Keywords:** carbapenemase, inhibitor, antibiotic resistance, carbapenem resistance, metallo-β-lactamases, serine-β-lactamases

## Abstract

Carbapenem resistance is a major global health problem that seriously compromises the treatment of infections caused by nosocomial pathogens. Resistance to carbapenems mainly occurs via the production of carbapenemases, such as VIM, IMP, NDM, KPC and OXA, among others. Preclinical and clinical trials are currently underway to test a new generation of promising inhibitors, together with the recently approved avibactam, relebactam and vaborbactam. This review summarizes the main, most promising carbapenemase inhibitors synthesized to date, as well as their spectrum of activity and current stage of development. We particularly focus on β-lactam/β-lactamase inhibitor combinations that could potentially be used to treat infections caused by carbapenemase-producer pathogens of critical priority. The emergence of these new combinations represents a step forward in the fight against antimicrobial resistance, especially in regard to metallo-β-lactamases and carbapenem-hydrolysing class D β-lactamases, not currently inhibited by any clinically approved inhibitor.

## 1. Introduction

β-lactams are the most diverse and widely used group of antibiotics in clinical practice. The mechanism of action of β-lactams is based on binding and blocking the penicillin binding proteins (PBPs), which are involved in the final steps of cell wall synthesis [1,2,3]. Carbapenems differ structurally from penicillins, cephalosporins and monobactams and have a wider spectrum of action and stability against β-lactamase enzymes. The longest established carbapenems are imipenem, meropenem and ertapenem, while more recently developed examples include doripenem, biapenem, panipenem, razupenem and tomopenem [4]. The use of carbapenems in clinical settings increased in the 2000s due to the emergence and spread of extended spectrum β-lactamases (ESBLs). However, the massive use/overuse of these agents led to the emergence of resistance, as had previously occurred with other groups of antibiotics [5].

In 2017 the World Health Organization (WHO) published a list of priority pathogens for which new treatments are required. The pathogens included in the highest category of urgency are carbapenem-resistant *Acinetobacter baumannii*, carbapenem-resistant *Pseudomonas aeruginosa* and carbapenem- and third generation cephalosporin-resistant *Enterobacterales* [6]. This situation highlights the importance of β-lactam antibiotics, especially carbapenems, in the treatment of infections caused by nosocomial pathogens. Production of carbapenemases (carbapenem-hydrolyzing enzymes) is the most important mechanism of carbapenem resistance [7], with examples in the four classes of β-lactamases, categorised according to the Ambler classification [8,9]. Among the carbapenemases identified, the following are the most important: (i) class A carbapenemases, especially those coded in plasmids, such as KPC and GES. The KPC group is the most widely distributed worldwide and the constituents are predominantly found in *Klebsiella pneumoniae*, although they have also been identified in *Enterobacter* spp., *Salmonella* spp., *P. aeruginosa* and *A. baumannii* [8,9,10]; (ii) class B carbapenemases (also known as metallo-β-lactamases, MBLs) are usually found in pathogens such as *P. aeruginosa*, *A. baumannii* and *Enterobacterales*, and their prevalence has increased in recent years [8,11]. The most common groups of MBLs are VIM, IMP and NDM [11]; (iii) class C carbapenemases are not numerous and have been identified recently. Although, class C β-lactamases production does not offer carbapenem resistance, exceptionally five enzymes in this group are capable of hydrolyzing carbapenems (ACT-1, DHA-1, CMY-2, CMY-10 and ADC-68) [9], and (iv) class D carbapenemases (also known as OXAs); although discovered many years ago, the rapid spread of carbapenem hydrolyzing class D β-lactamase (CHDLs) is recent [12,13]. OXA-48-like is widely disseminated in *Enterobacterales*, while the groups OXA-23-like, OXA-24/40-like, OXA-58-like, OXA-143-like and OXA-235-like are mainly responsible for resistance to carbapenems in *A. baumannii* [13,14] (Table 1).

Although carbapenemase activity is the main cause of carbapenem resistance, other elements are also involved. Porins have been shown to be associated with the development of resistance to carbapenems in synergy with hyperexpression of AmpC and/or ESBLs [15,16,17]. Similarly, efflux pumps [15] and mutations in PBPs (PBP2 or PBP3) have also been implicated in resistance to carbapenems [18,19]. 

One of the main strategies used to restore the effectiveness of β-lactam antibiotics is to use β-lactamase inhibitors (molecules that are able to bind to the active site of the enzyme) to prevent the antibiotic being hydrolyzed by the enzyme [20,21]. The first β-lactamase inhibitor discovered (in 1972) was clavulanic acid, followed by sulbactam (in 1978) and tazobactam (in 1984). These are the so-called classical β-lactamase inhibitors [20,21,22,23]. Clavulanic acid essentially inhibits class A β-lactamases, including ESBLs. Sulbactam displays less activity against class A β-lactamases than clavulanic acid or tazobactam, but is more effective against class C β-lactamases, and it also displays antimicrobial activity per se against *A. baumannii*. Tazobactam displays higher activity against CTX-M type enzymes (group within class A) than the others and is able to inhibit (very slightly) some class C and D β-lactamases [21,22,23]. The discovery and subsequent commercialization of these three inhibitors constitute “before” and “after” scenarios in antimicrobial therapy. However, the limited spectrum of inhibition (mainly class A, not including carbapenemases, and slight inhibition of class C β-lactamases) and the appearance and dissemination of new β-lactamases, particularly class B and D carbapenemases, led to the need to search for new inhibitors [2,24]. Fortunately, in recent years new groups of inhibitors have appeared, and some have already been approved by regulatory agencies and are now available in the clinical setting, thus extending and recovering the antimicrobial activity of some β-lactam antibiotics. The main new groups are diazobicyclooctanes (DBOs) (avibactam and relebactam have been approved by the FDA) and boronic acid derivatives (vaborbactam is currently the only inhibitor approved) [25,26]. Despite the development and commercialization of these new inhibitors, new compounds able to inactivate carbapenemases are still required. The limited therapeutic options—and sometimes the lack of any option—for strains carrying carbapenemases, maintains the resistance to β-lactams, particularly carbapenems, as one of the main current problems in the healthcare systems worldwide [27,28,29]. 

**Figure 1:**
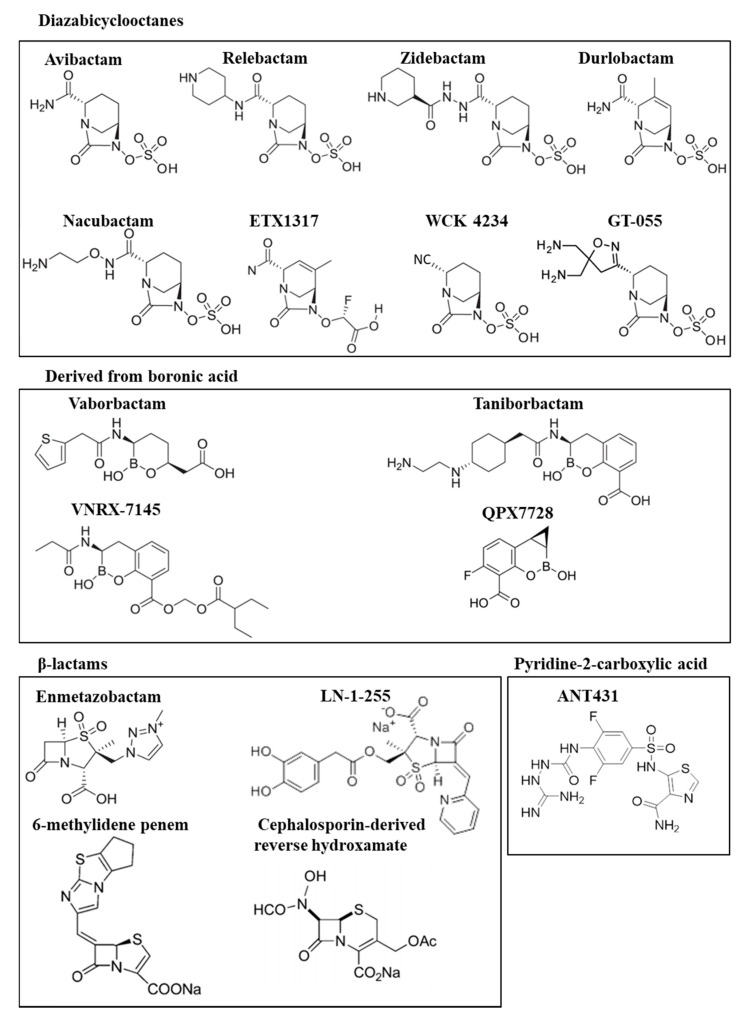
Structures of the recent carbapenemase inhibitors.

This review focuses on the recent studies of new carbapenemase inhibitors, recently approved for clinical use, or at preclinical or clinical stages of development (Figure 1). We consider those compounds capable of exhibiting high activity against the most widely distributed class A, B and D carbapenemases, with particular interest in boronic acid and DBO derivatives, two groups of new inhibitors that will be of key importance over the next decade in the development of inhibitors that will clear the way for the use of carbapenems.

## 2. Carbapenemase Inhibitors Recently Approved for Therapeutic Use

Efforts made in the last decade to develop new β-lactamase inhibitors able to protect β-lactams from the action of carbapenemases have led to the introduction in the clinical setting of three new, recently approved β-lactam/β-lactamase inhibitors: ceftazidime/avibactam, imipenem/relebactam and meropenem/vaborbactam. These three new combinations should be considered by clinicians as a real alternative treatment for infections caused by carbapenem-resistant pathogens, with demonstrated safety and efficacy.

To facilitate the description of the inhibitors included in this review, the main characteristics of these compounds are summarized in Table 2, Table 3, Table 4 and Table 5, including the clinical trials conducted (Table 2), the main published inhibition kinetics (IC_50_ or *K*_i app_, Table 3), the most important carbapenemases inhibited by each compound (Table 4) and finally, the main multi-drug resistant pathogens against which these new inhibitors could be used (Table 5).

### 2.1. Diazabicyclooctanes 

Diazacyclooctanes (DBOs) are a new family of non-β-lactam β-lactamase inhibitors which share a common five-membered diazabicyclooctane ring with an amide group that targets the active-site of serine β-lactamases via carbamylation [47]. In contrast to the findings on classical β-lactam-based inhibitors, DBOs do not undergo relevant structural rearrangement once the ring is opened, because of strong polar interactions with key conserved residues located in the vicinity of the active site. This particular mode of action results in extremely efficient inhibition, particularly of class A and C β-lactamases, although the effect is variable against class D enzymes [30,38,48,49,50]. There are currently two DBOs available for human use: avibactam and relebactam. 

#### 2.1.1. Avibactam (Ceftazidime/Avibactam)

The introduction of avibactam (formerly NXL104) in the clinical setting ended a 30-year dearth of new β-lactamase inhibitors in clinical practice. In sharp contrast to the narrow class-A-restricted inhibitory profile of classic β-lactam inhibitors, this first-in-class synthetic diazabicyclooctane exhibited an broad spectrum of activity against the most clinically relevant class A, C and D β-lactamases: KPC, GES, CTX-M, SHV, the chromosomal AmpCs of *P. aeruginosa* and *Enterobacter cloacae*, and, to a lesser extent, OXA-48 [51,52]. This wide spectrum of inhibition is based on a unique mechanism of action sustained by the particular structure of avibactam, which contains a five-membered diazabicyclooctane ring that targets the active site of the serine β-lactamase via a carbamylation reaction. This reaction occurs in a reversible manner and is followed by a slow deacylation route that releases intact avibactam. The released molecule can reacylate its β-lactamase target and initiate another cycle of inhibition [53]. Kinetic assays with purified protein extracts have demonstrated that avibactam exerts potent activity against KPC, OXA-48, CTX-M-like and *E. cloacae* and *P. aeruginosa* AmpC, all of which produce IC_50_ values in the nM range [31,54]. 

Developed in combination with ceftazidime (Zavicefta^®^, Pfizer Inc., NewYork, NY, USA), avibactam has been approved since 2015 for use to treat complicated urinary tract infections (cUTI), complicated Intra-abdominal Infections (cIAI)and hospital-acquired bacterial pneumonia/ (HABP)/ventilator-associated pneumonia (VAP) [55]. It is also being evaluated for other indications, e.g., in respiratory patients with cystic fibrosis (Clinicaltrial.gov identifier: NCT02504827) and pediatric patients with HABP (Clinicaltrial.gov identifier: NCT04040621). Among the recently approved β-lactam/β-lactamase inhibitor combinations, ceftazidime/avibactam shows the broadest spectrum of therapeutic coverage. Only metallo-β-lactamases and extended-spectrum OXA enzymes can escape its wide spectrum of activity. In vitro studies have demonstrated that the addition of avibactam restores the activity of ceftazidime against large collections of *Enterobacterales* that produce the most clinically relevant enzymes, such as KPC, OXA-48 and ESBLs [51,56]. Avibactam is also active against β-lactam resistant *P. aeruginosa*, including isolates with very high-levels of AmpC overexpression and OprD deficiency [57], although its activity against the non-fermenter appears to be lower than that of its counterpart ceftolozane/tazobactam, which is not reviewed here as it does not act against carbapenemase-producing organisms [58]. 

#### 2.1.2. Relebactam (Imipenem/Relebactam)

Relebactam (formerly MK-7655) is a novel DBO β-lactamase inhibitor that is closely related to its processor, avibactam. At the structural level, both inhibitors share an identical DBO core, which sustains a similar mechanism of β-lactamase inhibition. However, relebactam carries a piperidine ring at the 2-position carbonyl group that provides a positive charge to the molecule at physiological pH, which is key to preventing the extrusion of relebactam from bacterial cells [59]. It has a slightly more restricted spectrum of inhibition than avibactam as it does not display in vitro activity against OXA-48 enzymes (Table 3). However, it exhibits potent inhibitory activity against class A and C β-lactamases in vitro. Although relebactam shares the ability of avibactam to inactivate class C β-lactamases [60,61], recently published biochemical studies have reported that it is less able to block class A enzymes such as CTX-M-15 and KPC, probably due to unfavourable steric clashes between the relebactam piperidine ring and β-lactamase residues at positions 104 and 105 of CTX-M-15 and KPCs [36].

Relebactam was developed to potentiate the activity of imipenem (imipenem/cilastatin/relebactam) after displaying pharmacokinetic/pharmacodynamics (PK/PD) compatibility and effectiveness both in vitro and in mouse models of infection against carbapenem-resistant strains of *Enterobacterales* and *P. aeruginosa* [59,62]. More recently, the combination has proved to be effective and safe for the treatment of HABP/VAP caused by Gram-negative bacteria and for the treatment of HABP/VAP, cIAI, cUTI caused by bacteria not susceptible to imipenem in two phase III trials: RESTORE-IMI 1 [63] and RESTORE-IMI 2 [64] (Clinicaltrial.gov identifiers: NCT02452047 and NCT02493764, Table 2). The drug is now commercially available for the above-described indications under the brand name Recarbrio^®^ (Merck & Co., Kenilworth, NJ, USA).

Imipenem-relebactam is broadly active against a wide variety of Gram-negative pathogens, including *Enterobacterales*, *P. aeruginosa* and the anaerobic *Bacteroides* spp. [65,66]. More importantly, it has demonstrated excellent activity against key multidrug-resistant pathogens, for which it was intended to provide increased clinical coverage: (A) >82% susceptibility in isolates of *Enterobacterales* with difficult-to-treat phenotypes (resistance to all classical β-lactams and fluoroquinolones) [67]; (B) excellent activity against KPC- and ESBL-producing isolates from different species of *Enterobacterales*, including strains with porin alterations and international clones of *K. pneumoniae* and *E. coli* [68]; and (C) highly active against XDR *P. aeruginosa*, including imipenem-resistant strains showing carbapenem resistance due to OprD deficiency and against strains showing acquired resistance to ceftolozane/tazobactam and ceftazidime/avibactam due to production of GES-1, PER-1 and extended-spectrum OXA enzymes [69,70]. However, relebactam is not able to restore the activity of imipenem against strains bearing metallo-β-lactamases [71] or against isolates of *A. baumannii* producing horizontally-acquired class D carbapenemases (e.g., OXA-23, OXA-24/40 and OXA-58), thus indicating that, as with ceftazidime/avibactam, there remain some challenges to developing clinically available β-lactam/DBO combinations.

### 2.2. Boronic Acid Derivatives

In recent years special interest has arisen in developing non-acylating β-lactamase inhibitors. Although boron-based compounds were originally developed as serine-β-lactamases inhibitors, they have recently also been developed as metallo-β-lactamases inhibitors [72,73]. Cyclic boronates, which are more useful than the previously developed acyclic boronates, react rapidly with β-lactamases to form stable enzyme-inhibitor complexes. Thus, after early work on acyclic boronic acids, recent efforts have focused on cyclic boronates. 

The kinetic mechanisms of binding of bicyclic boronates to β-lactamases remain to be established; however, it has been observed that these compounds often display very good activity (IC_50_ below the mM range) [74,75]. The results of microbiological assays also support the potential of these compounds as broad spectrum β-lactamase inhibitors [76] (Table 3, Table 4 and Table 5).

### 2.3. Vaborbactam (Meropenem/Vaborbactam)

Vaborbactam (formerly RPX-7009) is the first clinically available cyclic boronate-based β-lactamase inhibitor. Developed with the aim of effectively inhibiting the epidemic class A carbapenemases KPC-2 and KPC-3, it also has a broad spectrum of activity which covers other “problematic” clinically-relevant class A (CTX-M-, SHV- and TEM-like) and C (DHA-, MIR-, FOX- and P99-like) β-lactamases that can confer resistance to broad-spectrum cephalosporins [77,78]. Currently available data on its kinetic parameters have shown that vaborbactam exhibits potent inhibitory activity against the aforementioned enzymes, for which IC_50_ values in the nM range have been obtained. However, it displays weak potency against class D β-lactamases (e.g., OXA-48), and it is totally inactive against class B enzymes [79] (Table 3).

Vaborbactam was initially approved for use in combination with meropenem by the FDA in August 2017, being the first carbapenem/β-lactamase inhibitor combination available for human use (Vabomere^®^, Menarini Group, Florencia, Italy). Active against *E. coli*, *K. pneumoniae* and *E. cloacae* complex, the efficacy and safety of meropenem/vaborbactam has been evaluated in two randomized clinical trials: targeting antibiotic non-susceptible Gram-negative organisms (TANGO) I (Clinicaltrial.gov identifier: NCT02166476) [80] and TANGO II (Clinicaltrial.gov identifier: NCT02168946) [81]. TANGO I demonstrated the efficacy of this new combination in cUTI, in comparison with piperacillin/tazobactam, whereas TANGO II demonstrated the efficacy of meropenem/vaborbactam in the treatment of infections (urinary tract infection, hospital-acquired pneumonia / ventilator-associated pneumonia, complicated intra-abdominal infections or bloodstream infection) caused by carbapenem-resistant Enterobacterales (CRE).

Microbiological studies have evaluated meropenem/vaborbactam in tests with large worldwide collections of KPC-producing isolates of *Enterobacterales*, and activity rates higher than 95% have been obtained in most cases [82,83,84]. Moreover, the addition of vaborbactam has also been shown to restore the wild type minimum inhibitory concentration (MIC) of meropenem in strains of *Enterobacterales* showing decreased meropenem susceptibility due to the production of AmpCs or ESBLs and reduced permeability [77]. However, vaborbactam is not able to improve the activity of meropenem against multidrug-resistant non-fermenting Gram-negative rods, and it thus shows very limited activity against *P. aeruginosa* and *Acinetobacter* species. 

### 2.4. Emerging Broad-Spectrum Resistance to Recently Approved β-Lactam/β-Lactamase Inhibitor Combinations Active against Carbapenemase-Producing/Carbapenem-Resistant Gram-Negative Pathogens

The introduction into the clinical setting of ceftazidime/avibactam, imipenem/relebactam and meropenem/vaborbactam provides a partial solution for some of the most clinically relevant carbapenemase-producing or carbapenem-resistant Gram-negative pathogens. Ceftazidime/avibactam provides the broadest range of therapeutic coverage, including against KPC, OXA-48 and carbapenem resistant organisms that produce ESBLs/AmpC and cause permeability defects. Imipenem/relebactam also exhibits strong activity against KPC-producing *Enterobacterales* and against *P. aeruginosa* strains showing carbapenem resistance due to OprD deficiency; however, the addition of relebactam does not improve the activity of imipenem against OXA-48 producers. Finally, meropenem/vaborbactam has the most restricted spectrum of activity, as vaborbactam inefficiently protects meropenem from OXA-48-mediated hydrolysis or from β-lactamase-independent mechanisms in *P. aeruginosa* [85]. 

Understanding the spectrum of activity of these new agents and the application of diagnostic methods that enable the rapid identification of the underlying resistance mechanisms in the target pathogen are key factors to therapeutic success, preventing the selection of resistant strains and extending the life of these recently approved combinations. As commented above, high levels of primary resistance to these agents are expected in areas where carbapenemase-producing *A. baumannii* strains are prevalent and/or where high resistance occur as a result of production of metallo-β-lactamases, which break down the target activity of avibactam, relebactam and vaborbactam. However, another factor of perhaps even greater importance is the emergence of resistance in susceptible strains during therapy. Since the approval of ceftazidime/avibactam in 2015, the development of resistance has been widely reported in multiple *Enterobacterales* and *P. aeruginosa* isolates [86,87]. In most cases, the resistance involves the acquisition of amino acid substitutions, insertions or deletions in chromosomal or horizontally acquired β-lactamases, leading to variants with enhanced cephalosporinase activity but impaired hydrolysis of other substrates, such as carbapenems [88,89,90]. KPC enzymes are probably the group of β-lactamases in which this phenomenon has been most extensively reported in *Enterobacterales*, particularly in *K. pneumoniae* [91,92]. Modification of either KPC-2 and KPC-3 variants is most frequently associated with this particular behaviour [54]. Other mechanisms involved in the emergence of resistance to ceftazidime/avibactam in *Enterobacterales* include the structural modification of CTX-M-like or AmpC enzymes [93], increased KPC expression in combination with inactivation of porins and enhanced AcrAB-TolC efflux [94]. Similar to observations in *Enterobacterales*, development of resistance to ceftazidime/avibactam during treatment of *P. aeruginosa* infections has mainly been associated with selection of variants of PDC-enzymes [95]. Selection of extended-spectrum OXA-2 or OXA-10 variants such as OXA-539, OXA-681 and OXA-14 have also been associated with in vivo acquisition of high-level ceftazidime/avibactam resistance and may also play an important role in areas where these enzymes may have become widespread [27,69,96]. In addition, the ceftazidime/avibactam-resistant AmpC or OXA variants selected during treatment of *P. aeruginosa* infections also always confer cross-resistance to the recently developed antipseudomonal ceftolozane/tazobactam, further limiting the choice of appropriate therapy [97].

Knowledge about the genetic events leading to the acquisition of resistance to imipenem-relebactam and meropenem-vaborbactam is much more limited than for ceftazidime-avibactam. Carbapenems are much more strongly affected than cephalosporins by β-lactamase-independent mechanisms that decrease their intracellular accumulation, such as decreased outer membrane permeability (e.g., OmpK35 and OmpK36 loss in *K. pneumoniae*) and enhanced AcrAB-TolC efflux [15]. Thus, the available data indicate that observed differences in the resistance patterns for ceftazidime/avibactam and carbapenem/β-lactamase inhibitors represent more a question of whether a carbapenem partner or a cephalosporin partner is used, rather than which β-lactamase inhibitor is used in the combination. Thus, in vitro selection experiments and analysis of clinical meropenem/vaborbactam resistant *K. pneumoniae* isolates have identified inactivating mutations in porins that may or may not be combined with increased *bla*_KPC_-copy number and enhanced AcrAB-TolC efflux [98]. A similar pattern has also been observed with imipenem/relebactam and *Enterobacterales*, in which accumulation of chromosomal mutations leading to decreased permeability has also been observed, although the mutations appear to have less impact on the MIC than for the meropenem/vaborbactam combination [68,99]. Finally, the precise mechanisms leading to development of imipenem/relebactam resistance in *P. aeruginosa* remains to be determined. Recent in vitro selection experiments with different imipenem/relebactam concentrations and the PAO1 reference strains and its Δ*mutS* hypermutator derivative have evidenced that acquisition of high-level resistance is only achieved in the mutator strain, with combinations of mutations leading to inactivation of OprD, modification of the imipenem target PBP1a and enhanced MexAB-OprM efflux [100]. However, the possible relevance of these mechanisms in clinical strains remains to be determined. 

As a whole, it seems clear that the potential emergence of resistance to all of these new combinations is far more dependent on selection of β-lactamase mutations leading to enhanced β-lactam hydrolysis, than on selection of mutations leading to resistance to inhibition. Nevertheless, judicious use of these agents and continuous surveillance in future years is encouraged to prevent the emergence and spread of resistance to these new combinations among target pathogens. 

## 3. New Carbapenemase Inhibitors in Development

### 3.1. Diazabicyclooctane Derived Inhibitors 

In the last few years the chemical scaffolds of the diazabicyclooctane class of β-lactamase inhibitors such as avibactam and relebactam have been modified to enhance the activity of these compounds [34]. Although DBOs do not exert inhibitory activity against class B β-lactamases, they can be combined with a monobactam, such as aztreonam, as these are stable to MBLs and need only to be protected from ESBL or AmpC enzymes [101]. Indeed, some second generation developmental DBO inhibitors, such as durlobactam, zidebactam and nacubactam are “dual action inhibitors” and have high affinity for the PBP2 of many Gram-negative species, thus exerting an antimicrobial effect. These DBOs thus exhibit “β-lactam enhancer” activity, potentiating partner β-lactams, and the new combination displays activity against carbapenemase-producing pathogens, including MBLs (Table 2, Table 3, Table 4 and Table 5). 

#### 3.1.1. Avibactam (Aztreonam/Avibactam)

Avibactam is capable of inhibiting ESBLs, AmpCs and the carbapenemases produced by *Enterobacterales*, such as KPC, GES, IMI, SME or OXA-48, but not MBLs [31,51,52]. A new combination is now being developed, aimed at MBLs, selecting the only monobactam approved to date, aztreonam.

Aztreonam evades the action of MBLs, for which it shows a low affinity [14,102], but it can be hydrolyzed by ESBLs and AmpC enzymes, which are frequently co-produced by most MBL-producing *Enterobacterales*. Thus, the aztreonam/avibactam combination has an advantage over the ceftazidime/avibactam combination in acting against class B carbapenemase-producing strains, a very interesting option which combines two drugs that have already been accepted for clinical use. Although it is a promising alternative against MBL-producing *Enterobacterales*, further studies are needed to determine its activity against MBL-producing *P. aeruginosa* strains [103,104]. Clinical cases with promising results have already been reported with the alternative combination of ceftazidime/avibactam with aztreonam to treat infections caused by strains carrying different classes of β-lactamases, including MBLs [105,106,107]. Another favourable aspect of this combination is that efficacy and safety of both compounds are well established. Two prospective randomized studies in clinical phase III are already underway to assess the efficacy, tolerability and safety of aztreonam/avibactam in the treatment of serious infection due to MBL-producing Gram-negative bacteria (Clinicaltrial.gov identifiers: NCT03580044 and NCT03329092, Table 2).

#### 3.1.2. Zidebactam (Cefepime/Zidebactam)

Zidebactam (formerly WCK 5107, Wockhardt, Mumbai, India), as well as other new generation DBOs such as nacubactam and durlobactam, inhibit PBPs, β-lactamases and display synergy with β-lactams [34]. Zidebactam is a new bicyclo-acyl hydrazide which displays activity against of Gram-negative bacteria such as *Enterobacterales*, *P. aeruginosa* and *A. baumannii*, including strains producing different class A, C and D β-lactamases, such as CTX-M-like, AmpC or OXA-48 [37,50,108,109]. Although zidebactam is derived from a DBO scaffold, it has been designed with the objective of augmenting PBP2 binding rather than increasing the β-lactamase-inhibitory activity of the compound, thus enhancing β-lactam activity; however, it also displays moderate activity as a β-lactam inhibitor [34]. Zidebactam in combination with cefepime is being developed as a treatment for infections caused by MDR bacteria, and several clinical trials have recently been developed (currently at phase I) to evaluate the safety, tolerability and pharmacokinetics of the combination. Previously designed avibactam showed a weak affinity for PBP2, thus limiting its activity to *Enterobacterales* [110]. However, zidebactam may be considered one of a new generation of DBO inhibitors with improved antimicrobial activity against *P. aeruginosa*; as this new antibacterial scaffold bypasses the classic mutation-driven *P. aeruginosa* resistance mechanisms.

#### 3.1.3. Durlobactam (Sulbactam/Durlobactam)

Durlobactam (previously ETX2514, Entasis Therapeutics, Waltham, USA), a diazabicyclooctenone β-lactamase inhibitor of class A, C and D β-lactamases [111], displays some intrinsic antimicrobial activity against some *Enterobacterales* [112]. It is being tested in combination with sulbactam, another β-lactamase inhibitor also able to inhibit class A and C β-lactamases. Sulbactam, commercially available in combination with ampicillin, displays antimicrobial activity against *Acinetobacter* spp. [113]. Although the precise mechanisms leading to sulbactam resistance in *A. baumannii* are not clear, the rates of resistance appear to have increased in recent years due to the emergence of OXA-type and AmpC enzymes [13,114]. Therefore, the new sulbactam/durlobactam combination is being developed for the treatment of infections caused by multidrug-resistant *A. baumannii*, including OXA-type carbapenemase-producing strains, with promising results. Durlobactam has shown to increase the in vitro susceptibility to sulbactam in different collections of clinical *A. baumannii* isolates [30,115,116,117]. Durlobactam is not active against class B carbapenemases such as NDM [117]. This inhibitor also restored imipenem activity against isogenic *P. aeruginosa* strains overexpressing carbapenemases such as KPC and OXA-48 [111]. A phase III clinical trial is currently being undertaken to evaluate the efficacy and safety of intravenous sulbactam-durlobactam, in combination with imipenem, to treat patients with infections caused by the *Acinetobacter baumannii-calcoaceticus* complex (Clinicaltrial.gov identifier: NCT03894046, Table 2).

#### 3.1.4. Nacubactam (Meropenem/Nacubactam)

Similarly to zidebactam, nacubactam (formerly RG6080/OP0595, Meiji Seika Pharma, Tokio, Japan; Fedora Pharmaceuticals, Alberta, Canada and Roche, Basilea, Switzerland) is a next generation DBO which also acts in different ways, by inhibiting class A and C β-lactamases, and as an antibiotic by inhibiting PBP2, thus enhancing the activity of the β-lactam partner [38]. It has a high affinity for class A carbapenemases such as KPC, but not for class D carbapenemases such as OXA-23 and OXA-24/40, displaying highest activity against class A β-lactamases such as TEM and CTX-M. To our knowledge, data on the inhibitory activity of nacubactam against OXA-48 are lacking. In combination with meropenem, nacubactam shows good synergy against carbapenem-resistant KPC-producing *Enterobacterales* [38,39]. In tests with a collection of MBL-producing *Enterobacterales*, meropenem/nacubactam and cefepime/nacubactam inhibited, respectively, 80.3% and 93.3% of MBL producers. The combination of both drugs inhibited 43.9% of 57 isolates highly resistant to nacubactam and meropenem when evaluated independently. Aztreonam/nacubactam, incorporating an MBL-stable β-lactam partner, was almost universally active against the MBL-producing *Enterobacterales*, displaying an enhancing effect [118]. Although in some in vitro studies, nacubactam did not increase the activity of meropenem against a collection of *P. aeruginosa* [119], meropenem-nacubactam has shown promising in vivo activity against meropenem-resistant KPC-producing *P. aeruginosa* in animal models, indicative of a potential role in the treatment of infections caused by this pathogen [120]. Nacubatam does not seem to display any antimicrobial activity against *A. baumannii* [119]. It has recently been reported that in non-fermenters nacubactam acts only as a β-lactamase inhibitor [121]. Two phase I clinical trials have been completed to determine the intrapulmonary concentrations of nacubactam and meropenem and to study the safety, tolerability and pharmacokinetics in healthy volunteers (Clinicaltrial.gov identifier: NCT02134834 and NCT03182504, Table 2).

#### 3.1.5. ETX1317 (Cefpodoxime/ETX1317) 

ETX1317 (Entasis Therapeutics) is a new DBO that displays broad spectrum activity against class A and C serine-β-lactamases (SBLs). The ester prodrug of ETX1317 is the compound ETX0282, used in combination with cefpodoxime (an oral third-generation cephalosporin), and it is currently in development for treatment of multidrug-resistant and carbapenem-resistant *Enterobacterales* infections. This combination exhibits good antimicrobial activity in *Enterobacterales* isolates with multiple β-lactamases, including KPC-producing types [40]. As the high polarity and low pK_a_ of DBOs are well-suited to intravenous administration, but lead to low oral bioavailability, the main advantage of ETX0282 is the oral dosing availability [122]. A phase I clinical trial evaluating the safety, tolerability and pharmacokinetics of ETX137 when administered orally to healthy individuals has recently been completed (Clinicaltrial.gov identifier: NCT03491748, Table 2) [122]. Although the compound displays affinity for the PBP2 of *E. coli*, the antimicrobial activity is possibly not as high as for zidebactam or durlobactam, and therefore β-lactam enhancement might not be as pronounced as in these antibiotics; however, the MIC_90_ of the cefpodoxime/ETX0282 combination is very low (0.013 mg/L) with large collections of *Enterobacterales* including those producing KPC, OXA-48 or MBLs carbapenemases [40]. As far as we are aware no studies on other MDR pathogens such as *P. aeruginosa* or *A. baumannii* have yet been published.

#### 3.1.6. WCK 4234 (Meropenem/WCK 4234) 

DBO inhibitors usually inhibit class A and C β-lactamases, do not inhibit metallo-β-lactamases and display variable behaviour against class D carbapenemases. However, they are generally not able to inhibit the OXA-type carbapenemases that cause most carbapenem resistance in *Acinetobacter* spp. [123]. WCK 4234 (Wockhardt) displays potent inhibitory activity against class A and D carbapenemases and class C enzymes. It does not show antibacterial activity, unlike other DBOs considered in this review. It can enhance the activity of carbapenems against *Enterobacterales* producing OXA-48 or KPC carbapenemases, but not against MBLs-producing *Enterobacterales*. Importantly, the meropenem-WCK 4234 combination displays activity against OXA carbapenemases produced by *A. baumannii*, such as OXA-23, OXA-24/40 and hyperproduced OXA-51. WCK4234 did not display any activity against a collection of carbapenem-resistant isolates of *P. aeruginosa* [34,123,124]. 

No clinical trials have yet been conducted with this compound; however, meropenem-WCK 4234 decreased the bacterial load of a multidrug-resistant OXA-23-producing *A. baumannii* strain by 2.5 log in murine models of neutropenic lung infection in preclinical trials [34].

#### 3.1.7. GT-055 (GT-1/GT-055)

The diazabicyclooctane inhibitor GT-055 (also referred to as LCB18-055, LegoChem Biosciences, Daejeon, Korea) exhibits intrinsic activity against many *Enterobacterales* isolates, which bind tightly to PBP2 [125]. It is being tested in combination with GT-1 (also known as LCB10-0200), a novel siderophore-dihydroxypyridone and a modified aminothiazolylglycyl cephalosporin, which exploits bacterial iron-uptake systems to enhance entry into Gram-negative pathogens using a “Trojan-horse” strategy. GT-055 is able to enhance the in vitro activity of GT-1 against many GT-1–resistant strains.

GT-055 is currently at early stages of development, and it is not yet clear whether it can inhibit carbapenemases; however, there is clearly some synergic action between these two compounds against carbapenem-resistant strains of *E. coli*, *K. pneumoniae* and *A. baumannii*, including carbapenemase-producing strains species, which probably contributes to its activity in combination with GT-1 against isolates of E. coli and K. pneumoniae. Preclinical trials are being developed to determine the pharmacokinetic-pharmacodynamic properties of GT-055 [126], and clinical assays are programmed to start in 2020 with the GT-1/GT-055 combination.

### 3.2. Boronic Acid Derived Inhibitors 

#### 3.2.1. Taniborbactam (Cefepime/Taniborbactam)

Taniborbactam (formerly VNRX-5133, Everest Medicines, New York, NY, USA) is a cyclic boronate with broad spectrum β-lactamase inhibitory activity against KPC, OXA-48 and MBLs (such as VIM and NDM, but not IMP) [42,127]. It was probably the first inhibitor showing direct inhibitory activity against Ambler class A, B, C, and D enzymes [41]. Taniborbactam uses distinct mechanisms to inhibit both SBLs and MBLs. It inhibits SBLs with slow dissociation, while in MBLs, it behaves as a reversible competitive inhibitor, with low inhibitor constant (*K_i_*) values and rapid dissociation [41].

Taniborbactam is being developed for use in combination with cefepime and meropenem to treat complicated infections caused by MDR pathogens such as carbapenem-resistant *Enterobacterales* and carbapenem-resistant *P. aeruginosa*, including strains expressing serine carbapenemases and metallo-β-lactamases. Several phase I clinical trials have been undertaken to evaluate its PK/PD and safety. A phase III clinical trial is being conducted with cefepime/taniborbactam to evaluate the safety and efficacy of this combination against complicated urinary tract infections, including infections caused by MBL-producing strains (registration No. NCT03840148 at ClinicalTrials.gov, Table 2).

#### 3.2.2. VNRX-5236 (Ceftibuten/VNRX-7145)

VNRX-7145 (VenatoRx Pharmaceuticals, Malvern, PA, USA) is a novel cyclic boronate β-lactamase inhibitor with good oral bioavailability. In vivo, VNRX-7145 undergoes biotransformation to the active VNRX-5236, which covalently and reversibly binds the active serine site of Ambler class A and D β-lactamases, including those that hydrolyze carbapenems, such as KPC and OXA-48 [128,129]. VNRX-7145 is being developed in combination with ceftibuten, because of the good oral bioavailability of the cephalosporin. The combination is mainly being developed to treat infections caused by carbapenemase- and ESBL-producing *Enterobacterales*. 

VNRX-5236 displays potent inhibition activity against class A (KPC-2) and D (OXA-48) β-lactamases, displaying IC_50_ in the nanoM range. However, it shows modest activity against the class B enzymes NDM-1 and VIM-2 (Table 3) [35,43,130]. A phase I clinical trial is currently underway to determine the safety and PK of this compound (registration No. NCT04243863 at ClinicalTrials.gov, Table 2).

#### 3.2.3. QPX7728 (Meropenem/QPX7728)

QPX7728 (QPEX Biopharma) is an ultra-broad-spectrum cyclic boronic acid β-lactamase inhibitor with activity against both SBLs and MBLs [31]. In comparison with other β-lactamase inhibitor combinations (such as meropenem-vaborbactam, ceftazidime-avibactam and imipenem-relebactam), meropenem plus QPX7728 was found to be the most potent β-lactam-β-lactamase inhibitor combination tested against all groups of carbapenem resistant *Enterobacterales* with multiple resistance mechanisms, including KPC and MBLs carbapenemases [131]. Similarly, the meropenem/QPX7728 combination showed antimicrobial activity against a collection of carbapenem-resistant *A. baumannii* isolates producing CHDLs, NDM and KPC carbapenemases [131], and it was also being active against KPC-producing *P. aeruginosa* strains [132]. This inhibitor exhibits excellent affinity for important carbapenemases (KPC-2, IMP-1, VIM-1, NDM-1, OXA-23 and OXA-48), with *K*_i_ values in the low nM range (Table 3). In preclinical assays, this inhibitor greatly increased the efficacy of several β-lactams against KPC-producing *Enterobacterales* in a neutropenic mouse thigh infection model [133]. QPX7728 is orally bioavailable, and combinations with ceftibuten and tebipenem have been tested with carbapenem- resistant *Enterobacterales* [134].

A phase I clinical trial has recently begun to evaluate the safety and pharmacokinetics of intravenous treatments with QPX7728 (registration No. NCT04380207 at ClinicalTrials.gov, Table 2).

### 3.3. β-Lactam-Derived Inhibitors. Penicillin Sulfones

Penicillin-based sulfones, such as clavulanic acid, sulbactam and tazobactam, were the first inhibitors to be discovered. Unfortunately, none of these three compounds can inhibit carbapenemases. In recent years, within the group of penicillin sulfones, several molecules have emerged with higher or broader activity than the classical inhibitors, but only enmetazobactam and LN-1-255 have exhibited a relevant activity against carbapenemases.

#### 3.3.1. Enmetazobactam (Cefepime/Enmetazobactam)

Enmetazobactam (formerly AAI101, Allecra Therapeutics, Wallbrunnstr, Germany), a novel penicillanic acid sulfone ESBLs inhibitor, also displays slight activity against some class C and D carbapenemases and also against some class A carbapenemases from *Enterobacterales* strains [135,136,137,138]. Enmetazobactam differs from tazobactam in the presence of a methyl group in the triazole moiety, which causes the compound to have a net neutral charge that enhances its activity [44]. The cefepime/enmetazobactam combination produced better results against MDR ESBLs-producing *Enterobacterales* strains than recently approved treatments such as ceftazidime/avibactam, ceftolozane/tazobactam and imipenem/relebactam [139]. Enmetazobactam is a good inhibitor of all class A β-lactamases, including the carbapenemases KPC-2 and KPC-3, with IC_50_ values in the nanomolar range, and it presents higher levels of inhibition than tazobactam and similar levels to those displayed by avibactam. However, the levels of inhibition observed against class C and D are significantly lower for enmetazobactam than for the other two compounds (Table 3) [44]. The cefepime/enmetazobactam combination exerted higher activity than cefepime alone against carbapenemase-producing *Enterobacteriaceae* in an in vivo mice model [140]. The safety, tolerability and pharmacokinetics of cefepime/enmetazobactam have been established, and this combination was recently tested in a phase III trial in patients with complicated urinary infections or acute pyelonephritis (registration No. NCT03687255 at ClinicalTrials.gov, Table 2).

#### 3.3.2. LN-1-255 (Imipenem or Meropenem/LN-1-255)

A group of penicillin sulfones with activity against class A, C and D β-lactamases have been synthesized by John D. Buynak and collaborators [141,142,143]. Among these, the LN-1-255 molecule is the most widely studied due to its ability to inhibit CHDLs, yielding good results in combination with carbapenems. LN-1-255 displayed a significant ability to inhibit the carbapenemases typically produced by *A. baumannii* (OXA-23, OXA-24/40, OXA-51, OXA-58, OXA-143 and OXA-235) and the OXA-48 carbapenemase produced by *Enterobacterales* (Table 3) [33,45,144,145]. LN-1-255 possesses a catechol group that is responsible for its effectiveness due to its ability to enhance internalization of the compound in the bacteria through iron uptake systems [26]. In vivo models of murine pneumonia showed a reduction in the bacterial load in mice treated with imipenem/LN-1-255, of between 1.7–4.5 logs, in lungs infected with CHDL-producing *A. baumannii* strains [146]. In 2020, Rodriguez and collaborators designed a series of six 6-arylmethylidene penicillin-based sulfones, derived from LN-1-255, through modifications in the pyridine ring. Some of these compounds improved the efficiency of action against CHDLs [147]. 

### 3.4. Other Promising MBL Inhibitors 

MBLs are Zn(II)-dependent enzymes with the ability to hydrolyze β-lactam antibiotics. No clinically useful inhibitors of these enzymes have yet been approved. In the last few years, new structures focused specifically on inhibiting MBLs have been developed, with the compound ANT2681 (Antabio) probably representing one of the most important examples. This novel thiazole-carboxylate inhibitor, optimized from ANT431, is the result of a medicinal chemistry hit-to-lead program starting with pyridine-2-carboxylic acid, and it is a preclinical candidate with potential for clinical development as a specific inhibitor of MBLs [46]. ANT2681 inhibits the activity of MBLs through interaction with the dinuclear zinc ion cluster present in the active site of these enzymes [148]. The inhibitor displays the highest affinity for NDM-1, lower affinity for VIM-1 and very poor affinity for IMP-1. This inhibitor has shown efficacy in a mouse thigh model with an NDM-1-producing clinical isolate of *K. pneumoniae*. Although meropenem was ineffective at reducing tissue burden, its coadministration with ANT2681 yielded a statistically significant (1.8 log) reduction in colony forming units. Thus, ANT2681 is undergoing preclinical development with the intention of combining it with meropenem as a new treatment for serious infections caused by MBL-producing CRE.

In addition to the above-mentioned β-lactam-derived inhibitors, other compounds with a β-lactam core are capable of inhibiting carbapenemases, but which are at early stages of development. Thus, within the group of penicillin sulfones, C-6 substitutions affect the specificity of inhibition, displaying good activity against some class B β-lactamase [149]. In the group of penems, fusion of different bicyclic and tricyclic heterocycles with 6-methylidine penem yielded activity against class B carbapenemases [150,151,152]. CcrA and IMP-1, among others, were inhibited by J-110,441, a 1β-methylcarbapenem with a benzothenyl moiety at the C-2 position [153]. J-111,225, another new 1β-methylcarbapenem (with a trans-3,5 disubstituted pyrrolidinylthio moiety in C-2), inhibited IMP-1 [154] and showed bactericidal activity per se against *S. aureus* and *P. aeruginosa* [155]. Reverse hydroxamates (cephalosporins derived molecules) have exhibited activity against the MBL GIM-1 [156], or bisthiazolidines, also able to inhibit MBL enzymes [157]. 

Fragment-based drug discovery is also being used to develop a new class of inhibitors of dipicolinic acid derivatives in the fight against MBLs [158]. Other interesting inhibitors being developed against MBLs, whose efficacy remains to be demonstrated in preclinical trials, include triazole inhibitors [159]. Several recent excellent papers have reviewed many of these and other new inhibitory compounds at early stages of development, especially in relation to MBLs [160,161,162,163,164,165,166].

## 4. Major Challenges in the Development of New Carbapenemase Inhibitors

As discussed above, a new generation of carbapenemase inhibitors is being developed. Development of inhibitors of MBL type and *A. baumannii* CHDL enzymes is perhaps the most difficult challenge. One of the main difficulties in designing inhibitors of class B β-lactamases is the wide genetic diversity among these enzymes. Thus, e.g., taniborbactam can inhibit NDM and VIM but not IMP enzymes. On the other hand, small molecules able to bind and chelate zinc ions have been reported to inhibit MBLs; however, they also inhibit human metalloenzymes and they may therefore be toxic to living tissues. Preclinical assays are also complicated to perform, due to the lack of zinc needed for appropriate behaviour of the MBLs at the infection sites [11,167]. In vitro conditions used for determining antibiotic susceptibility are very different from those that actually occur during infection [168]. It is therefore challenging to design and evaluate specific inhibitors for MBLs, and further research is necessary. 

CHDLs, especially those produced by *A. baumannii*, are resistant to the action of most classical inhibitors [13]. The moderate capacity of CHDLs to hydrolyse carbapenems, combined with low permeability of *A. baumannii*, generates a high level of resistance to these antibiotics, which are considered the first choice for treating *A. baumannii*. There is an urgent need to develop new compounds capable of restoring the susceptibility to carbapenems in CHDL-producing strains of *A. baumannii*. So far, two compounds have exhibited useful activity against these enzymes: durlobactam [30] and LN-1-255 [33]. In both cases the key factor was the development of highly permeable compounds. Another important challenge is the coexistence of different β-lactams in the same pathogen. Thus, it is common to find numerous β-lactamases in *Enterobacterales*, for example strains co-producing *bla_NDM-1_*, *bla_OXA-48_*, *bla_CTX-M-15_*, *bla_TEM-1_* and *bla_SHV-182_* [169], often expressing two or more different carbapenemases or even MBLs.

Future inhibitors must be very potent and able to inhibit different classes of β-lactamases at the simultaneously, which requires complex structural and biochemical development [170]. Likewise, research must continue in order to develop new compounds that are effective against the main enzymatic resistance mechanism of this multi-drug resistant pathogen.

## 5. Final Considerations 

Carbapenems are the most effective of the β-lactam antibiotics and display a broad spectrum of antibacterial activity. Their molecular structure, including a carbapenem together with the β-lactam ring, provides great stability against hydrolysis of most β-lactamases. These compounds are therefore used as the most appropriate last-resort treatment against severe infections. Moreover, they do not cause many adverse effects. For these reasons, carbapenem resistance is a public-health problem of global dimensions.

Carbapenem resistance, mainly mediated by carbapenemases, dramatically limits treatment options for infections caused by Gram-negative bacteria, which are resistant to carbapenems and also to most β-lactams. Unfortunately, these pathogens often have genetic determinants of resistance to other antibiotics such as aminoglycosides and quinolones. They are therefore often only susceptible to antibiotics such as fosfomycin and colistin, which have different problems associated with toxicity and effectiveness; tigecycline is the only rescue therapy available [171], and resistance to this drug is rapidly increasing [172].

Although the introduction of these new carbapenemase inhibitors has changed the clinical scenario, adequate antimicrobial stewardship programmes and carbapenem sparing strategies are required in clinical settings to preserve the effectiveness of these antibiotics [173]. The rational use of carbapenems as well as appropriate measures of infection control and prevention are essential to minimize the misuse and overuse of these antibiotics in an attempt to prevent the spread of carbapenem resistance and prolong the time during which these new inhibitory molecules will remain effective.

While we depend on carbapenems as the main option against resistant pathogens, what will possibly happen for at least during the next decade, we must continue to develop compounds with the capacity to inhibit carbapenemase enzymes, which have become the major obstacle to treating serious infections caused by multi-drug resistant pathogens.

## Figures and Tables

**Table 1:** Most clinically significant carbapenemases.

Mechanism of Action	Class	Carbapenemase	More Common Enzymes			
Serine-β-lactamases	A	KPC	KPC-2	KPC-3		
		GES	GES-2	GES-5	GES-6	
	D	OXA	OXA-23	OXA-24/40	OXA-58	OXA-48
Metallo-β-lactamases	B	IMP	IMP-1	IMP-6	IMP-7	
		VIM	VIM-1	VIM-2		
		NDM	NDM-1	NDM-4	NDM-5	

**Table 2:** Carbapenemase inhibitors undergoing clinical trials.

Antibiotic/Inhibitor Combination	ClinicalTrials.gov Identifier	Phase	Title	Status	Start Date
**Diazabicyclooctane-derived**					
Ceftazidime/Avibactam	NCT04040621	I	Single-dose PK Study of Ceftazidime-Avibactam in Hospitalized Children Receiving Systemic Antibiotics for Nosocomial Pneumonia	Recruiting	June, 2020
Ceftazidime/Avibactam	NCT02504827	IV	Steady-State Pharmacokinetics of Ceftazidime/Avibactam in Cystic Fibrosis	Completed	September, 2015
Imipenem /Relebactam	NCT02452047	III	Efficacy and Safety of Imipenem + Cilastatin/Relebactam (MK-7655A) Versus Colistimethate Sodium+Imipenem+Cilastatin in Imipenem-Resistant Bacterial Infection (MK-7655A-013)	Completed	September, 2017
Imipenem /Relebactam	NCT02493764	III	Imipenem/Relebactam/Cilastatin Versus Piperacillin/Tazobactam for Treatment of Participants with Bacterial Pneumonia (MK-7655A-014)	Completed	April, 2019
Meropenem/Vaborbactam	NCT02166476	III	Efficacy/Safety of Meropenem-Vaborbactam Compared to Piperacillin-Tazobactam in Adults with cUTI and AP	Completed	November, 2014
Meropenem/Vaborbactam	NCT02168946	III	Efficacy, Safety, Tolerability of Vabomere Compared to Best Available Therapy in Treating Serious Infections in Adults	Completed	July, 2014
Aztreonam/Avibactam	NCT01689207	I	To Investigate the Safety and Tolerability of Aztreonam-Avibactam (ATM-AVI)	Completed	September, 2012
Aztreonam/Ceftazidime/Avibactam	NCT03978091	I	A Trial to Evaluate the Pharmacokinetics and Safety of AVYCAZ(R) in Combination with Aztreonam	Recruiting	June, 2019
Aztreonam/Avibactam	NCT04486625	I	Pharmacokinetic Study of Aztreonam-Avibactam in Severe Renal Impairment	Recruiting	August, 2020
Aztreonam/Avibactam	NCT02655419	II	Determine the PK and Safety and Tolerability of ATM-AVI for the Treatment of cIAIs in Hospitalized Adults (REJUVENATE)	Completed	May, 2016
Aztreonam/Avibactam/Metronidazole	NCT03329092	III	A Study to Determine the Efficacy, Safety and Tolerability of Aztreonam-Avibactam (ATM-AVI) ± Metronidazole (MTZ) Versus Meropenem (MER) ± Colistin (COL) for the Treatment of Serious Infections due to Gram-Negative Bacteria (REVISIT)	Recruiting	April, 2018
Aztreonam/Avibactam	NCT03580044	III	Efficacy, Safety, and Tolerability of ATM-AVI in the Treatment of Serious Infection Due to MBL-producing Gram-negative Bacteria	Not yet recruiting	December, 2020
Zidebactam	NCT02674347	I	MAD Study to Evaluate the Safety, Tolerability and Pharmacokinetics of Intravenous Zidebactam in Healthy Adults	Completed	February, 2016
Cefepime/Zidebactam	NCT02707107	I	MED Study to Evaluate the Safety, Tolerability and Pharmacokinetics of Intravenous WCK 5222 (Zidebactam and Cefepime) in Healthy Volunteers	Completed	March, 2016
Cefepime/Zidebactam	NCT02942810	I	To Investigate the Pharmacokinetics of Intravenous WCK 5222 (FEP-ZID) in Patients with Renal Impairment	Completed	October, 2016
Cefepime/Zidebactam	NCT03554304	I	Evaluate the Effect of WCK 5222 on the QT/QTc Interval in Healthy Volunteers	Completed	February, 2017
Cefepime/Zidebactam	NCT03630094	I	Plasma and Intrapulmonary Concentrations Study of WCK 5222	Completed	March, 2017
Cefepime/Zidebactam	NCT02532140	I	Study to Evaluate the Safety, Tolerability, and Pharmacokinetics of WCK 5107 Alone and in Combination with Cefepime	Completed	August, 2017
Durlobactam	NCT02971423	I	Evaluation of the Safety, Tolerability and Pharmacokinetics of Intravenous ETX2514 Administered in Healthy Subjects	Completed	October, 2016
Durlobactam	NCT03985410	I	Study Evaluating the Effect of ETX2514 on Cardiac Repolarization in Healthy Male or Female Volunteers	Completed	May, 2019
Durlobactam	NCT04018950	I	Study to Determine the Excretion and Metabolism of 14C-ETX2514 Administered Intravenously in Healthy Male Subjects	Completed	June, 2019
Sulbactam/Durlobactam	NCT03303924	I	Study to Determine and Compare Plasma and Intrapulmonary Concentrations of ETX2514 and Sulbactam in Healthy Subjects	Completed	August, 2017
Sulbactam/Durlobactam	NCT03310463	I	Evaluation of the Pharmacokinetics, Safety, and Tolerability of Intravenous ETX2514 and Sulbactam Administered Concurrently to Subjects with Various Degrees of Renal Impairment and Healthy Matched Control Subjects	Completed	October, 2017
Sulbactam/Durlobactam	NCT03445195	II	Evaluation of Safety and Efficacy of Intravenous Sulbactam-ETX2514 in the Treatment of Hospitalized Adults with Complicated Urinary Tract Infections	Completed	January, 2018
Sulbactam/Durlobactam/Imipinem/Cilastatin	NCT03894046	III	Study to Evaluate the Efficacy and Safety of Intravenous Sulbactam-ETX2514 in the Treatment of Patients with Infections Caused by *Acinetobacter bumannii-calcoaceticus* Complex (ATTACK)	Recruiting	April, 2019
Nacubactam	NCT02134834	I	A Phase I Study to Assess Safety, Tolerability and Pharmacokinetics of OP0595	Completed	May, 2014
Meropenem/Nacubactam	NCT03182504	I	A Study to Investigate the Intrapulmonary Lung Penetration of Nacubactam in Healthy Participants	Completed	June, 2017
ETX0282	NCT03491748	I	A Study to Evaluate the Safety, Tolerability, and Pharmacokinetics (PK, the Measure of How the Human Body Processes a Substance) of ETX0282 when Administered Orally to Healthy Participants	Completed	March, 2018
**Boronic acid-derived**					
Taniborbactam	NCT02955459	I	VNRX-5133 SAD/MAD Safety and PK in Healthy Adult Volunteers	Completed	November, 2016
Cefepime/Taniborbactam	NCT03332732	I	VNRX-5133 Drug–Drug Interaction in Healthy Adult Volunteers	Completed	October, 2017
Cefepime/Taniborbactam	NCT03690362	I	VNRX-5133 with VNRX-5022 in Subjects with Varying Degrees of Renal Impairment	Completed	April, 2018
Cefepime/Taniborbactam	NCT03870490	I	Safety and Pharmacokinetics of VNRX-5133 in the Epithelial Lining Fluid of Healthy Adult Subjects	Completed	March, 2019
Cefepime/Taniborbactam	NCT03840148	III	Safety and Efficacy Study of Cefepime/VNRX-5133 in Patients with Complicated Urinary Tract Infections	Recruiting	August, 2019
VNRX-5236	NCT04243863	I	VNRX-7145 SAD/MAD Safety and PK in Healthy Adult Volunteers	Recruiting	January, 2020
QPX7728	NCT04380207	I	P1 Single and Multiple Ascending Dose (SAD/MAD) Study of IV QPX7728 Alone and Combined with QPX2014 in NHV	Not yet recruiting	November, 2020
**β-lactam-derived**					
Enmetazobactam	NCT03775668	I	Single Dose Mass Balance Study with C14—Labeled AAI101 in Healthy Male Volunteers	Completed	November, 2018
Cefepime or Piperacillin/Enmetazobactam	NCT03685084	I	Investigation of AAI101 Safety, Tolerability and PK in Healthy Volunteers	Completed	October, 2013
Cefepime/Enmetazobactam	NCT03680378	I	Lung Pharmacokinetics (PK) in Epithelial Lining Fluid (ELF)	Completed	July, 2017
Cefepime/Enmetazobactam	NCT03680352	I	Pharmacokinetics of Cefepime and AAI101 in Subjects with Renal Insufficiency and Healthy Subjects	Unknown	September, 2017
Cefepime/Enmetazobactam	NCT03680612	II	Cefepime/AAI101 Phase 2 Study in Hospitalized Adults with cUTI	Completed	September, 2017
Cefepime/Enmetazobactam	NCT03687255	III	Safety and Efficacy Study of Cefepime-AAI101 in the Treatment of Complicated Urinary Tract Infections	Completed	September, 2018

**Table 3:** Kinetic parameters of compounds tested against main carbapenemases.

Inhibitor		Clinically Most Relevant Carbapenemases						
		KPC-2	IMP-1	VIM-1	NDM-1	OXA-23	OXA-24/40	OXA-48
**Diazabicyclooctane-derived**								
**Avibactam**	**IC_50_ (nM)**	170 [30] */22 [31]/60 [32]	>1.6 × 10^5^ [31]/>1.0 × 10^5^ [32]	>1.6 × 10^5^ [31]	>1.6 × 10^5^ [31]/>1.0 × 10^5^ [32]	3.1 × 10^3^ [31]/8.9 × 10^3^ [33]	1.60 × 10^4^ [30]/2.23 × 10^4^ [33]	180 [31]/550 [32]
	***K_i_*_app_ (nM)**	900 [34]/11 [35]	>4.0 × 10^4^ [35]	>4.0 × 10^4^ [35]	>4.0 × 10^4^ [35]	>1.0 × 10^5^ [34]/1.7 × 10^3^ [35]	>1.0 × 10^5^ [34]/1.54 × 10^5^ [33]	3.0 × 10^4^ [34]/27 [35]
**Relebactam**	**IC_50_ (nM)**	82 [31]/230 [36]	>1.6 × 10^5^ [31]	>1.6 × 10^5^ [31]	>1.6 × 10^5^ [31]			9 × 10^4^ [31]
	***K_i_*_app_ (nM)**	2.2 × 10^3^ [34]/1.2 × 10^3^ [36]				>1.0 × 10^5^ [34]	>1.0 × 10^5^ [34]	>1.0 × 10^5^ [34]
**Zidebactam**	**IC_50_ (nM)**							
	***K_i_*_app_ (nM)**	4.5 × 10^3^ [34]		>1.00 × 10^5^ (VIM-2) [37]		>1.0 × 10^5^ [34]	>1.0 × 10^5^ [34]	>1.0 × 10^5^ [34]
**Durlobactam**	**IC_50_ (nM)**	4 [30]					190 [30]	
	***K_i_*_app_ (nM)**							
**Nacubactam**	**IC_50_ (nM)**	869 [38]	>3.0 × 10^5^ [38]			4.64 × 10^4^ [38]		
	***K_i_*_app_ (nM)**	3.1 × 10^4^ [39]						
**ETX1317**	**IC_50_ (nM)**	43 [40]					540 [40]	77 [40]
	***K_i_*_app_ (nM)**							
**WCK 4234**	**IC_50_ (nM)**							
	***K_i_*_app_ (nM)**	320 [34]				8.0 × 10^3^ [34]	5.0 × 10^3^ [34]	290 [34]
**Boronic acid-derived**								
**Vaborbactam**	**IC_50_ (nM)**	110 [31]/100 [32]	>1.6 × 10^5^ [31]/>1.0 × 10^5^ [32]	>1.6 × 10^5^ [31]	>1.6 × 10^5^ [31]/>1.0 × 10^5^ [32]	1.2 × 10^5^ [31]		6.9 × 10^3^ [31]/3.88 × 10^4^ [32]
	***K_i_*_app_ (nM)**	22 [41]/56 [35]	>3.0 × 10^4^ [41]/>4.0 × 10^4^ [35]	>4.0 × 10^4^ [35]	>3.0 × 10^4^ [41]/>4.0 × 10^4^ [35]	>4.0 × 10^4^ [35]		350 [41]/1.4 × 10^4^ [35]
**Taniborbactam**	**IC_50_ (nM)**	30 [32]	2.51 × 10^3^ [42]/3.98 × 10^4^ [32]	7.9 [42]	10 [42]/190 [32]			537 [42]/420 [32]
	***K_i_*_app_ (nM)**	4 [41]	>3.0 × 10^4^ [41]	19 (VIM-2) [41]	81 [41]			350 [41]
**VNRX-5236**	**IC_50_ (nM)**	80 [43]	>1.0 × 10^5^ [43]	9.04 × 10^3^ (VIM-2) [43]	3.81 × 10^4^ [43]			317 [43]
	***K_i_*_app_ (nM)**	110 [43]						
**QPX7728**	**IC_50_ (nM)**	2.9 [31]	610 [31]	14 [31]	55 [31]	1.2 [31]		1.1 [31]
	***K_i_*_app_ (nM)**	1.9 [35]	220 [35]	8 [35]	32 [35]	0.74 [35]		0.28 [35]
**β-lactam-derived**								
**Enmetazobactam**	**IC_50_ (nM)**	360 ^a^ [44]						1.1 × 10^4 a^ [44]
	***K_i_*_app_ (nM)**							
**LN-1-255**	**IC_50_ (nM)**					12 [33]	15 [33]	3 [45]
	***K_i_*_app_ (nM)**					88 [33]	289 [33]	170 [45]
**Pyridine-2-carboxylic acid**								
**ANT2681**	**IC_50_ (nM)**							
	***K_i_*_app_ (nM)**		3.81 × 10^3^ [46]	630 [46]	40 [46]			

^a^ Assays performed with bacterial extracts, not purified enzyme. * Numbers in brackets corresponde with references.

**Table 4:** Inhibition of major carbapenemases by new carbapenemase inhibitors.

**Inhibitor**	**Carbapenemase**						
	Class A	Class B			Class D		
	KPC	NDM	VIM	IMP	OXA-23	OXA-24/40	OXA-48
**Diazabicyclooctane-derived**							
Relebactam	✓	✗	✗	✗	✗		✗
Avibactam	✓	✗	✗	✗	✗	✗	✓
Zidebactam	✓ ^a^	✗	✗	✗	✗	✗	✗
Durlobactam	✓	✗		✗	✓	✓	✓
Nacubactam	✓ ^b^	✗	✗	✗	✗	✗	
ETX1317	✓						✓
WCK 4234	✓	✗	✗	✗	✓ ^c^	✓ ^d^	✓
**Boronic acid derived**							
Vaborbactam	✓	✗	✗	✗	✗		✗
Taniborbactam	✓	✓	✓	✗			✓
VNRX-5236	✓ ^e^	✗	✗	✗			✓
QPX7728	✓	✓	✓	✓	✓		✓
**β-lactam-derived**							
Enmetazobactam	✓						✓ ^f^
LN-1-255					✓	✓	✓

✓, useful inhibitory activity experimentally demonstrated; ✗, non-inhibition or not useful inhibitory activity demonstrated; blank square, not evaluated. Intermediate inhibition activity: IC_50_ or *K_i_* values: ^a^ K_i_ = 4.5 µM, ^b^ IC_50_ = 0.87 µM, ^c^ K_i_ = 8 µM, ^d^ K_i_ = 5 µM, ^e^ IC_50_ = 5.2 µM and ^f^ IC_50_ = 1.1 µM.

**Table 5:** Potential therapies aimed at treating infections caused by priority carbapenem-resistant pathogens for which new drugs are urgently needed.

New B-lactam/B-lactamase Inhibitor	Main Bacterial Targets					
	Carbapenem Resistant *A. Baumannii*		Carbapenem Resistant *P. Aeruginosa*		Carbapenem Resistant *Enterobacterales*	
	SBLs	MBLs	SBLs	MBLs	SBLs	MBLs
**Diazabicyclooctane derived inhibitors**						
Ceftazidime/avibactam			✓		✓	
Imipenem/relebactam						
Aztreonam/avibactam					✓	✓
Cefepime/zidebactam			✓	✓	✓	✓
Sulbactam/durlobactam	✓					
Meropenem(or cefepime, or aztreonem)/nacubactam			✓		✓	✓
Cefpodoxime/ETX1317					✓	✓
Meropenem/WCK 4234	✓	✗			✓	✗
GT-1/GT-055 ^a^	✓	✓ ^b^			✓	✓
**Boronic acid derivative inhibitors**						
Meropenem/vaborbactam						
Cefepime (or meropenem)/ taniborbactam			✓	✓	✓	✓
VNRX-7145/ceftibuten					✓	
Meropenem/QPX7728	✓	✓	✓	✓	✓	✓
**β-lactam-derived inhibitors**						
Cefepime/Enmetazobactam					✓	
Imipenem/LN-1-255	✓				✓	

^a^ preliminary results: efficacy due to the new siderophore-cephalosporin GT-01, rather than to the inhibitor GT-055. ^b^ synergy in carbapenem resistant IMP-producing *A. baumannii* strain.
